# Effects of Dietary NFC/NDF and Allicin Supplementation on Serum Electrolytes, Nitrogen and Phosphorus Excretion, and Fecal Microbiota in Beef Bulls

**DOI:** 10.3390/ani16132080

**Published:** 2026-07-05

**Authors:** Min Fu, Jinwen Lai, Wen Liu, Xu Zhang, Tianbao Chen, Zhisheng Wang

**Affiliations:** 1 Animal Nutrition Institute, Sichuan Agricultural University, Chengdu 611130, China; 2025114044@stu.sicau.edu.cn; 2 Sichuan Animal Sciences Academy, Chengdu 610066, China; 18380427514@163.com (J.L.); liuwen37@scsaas.cn (W.L.); zhangxu_bjtu@163.com (X.Z.); 3 Animal Genetic Breeding and Reproduction Key Laboratory of Sichuan Province, Chengdu 610066, China

**Keywords:** beef bulls, non-fibrous carbohydrate to neutral detergent fiber ratio, allicin, urinary parameters, nitrogen excretion, fecal microbiota

## Abstract

This study evaluated the effects of dietary non-fiber carbohydrate to neutral detergent fiber ratio (NFC/NDF) and allicin supplementation on serum electrolytes, urinary biochemical indicators, nitrogen (N) and phosphorus excretion, and fecal microbiota in beef bulls. Forty 12-month-old beef bulls were assigned to a 2 × 2 factorial design based on dietary NFC/NDF (0.75 and 1.02) and allicin supplementation (0 and 0.04% on a DM basis), resulting in four treatment groups with 10 replicates per group. Increasing dietary NFC/NDF decreased serum pH and increased the anion gap (*p* < 0.05). An interaction between NFC/NDF and allicin supplementation was observed for the serum concentrations of non-ionized calcium and total calcium (*p* < 0.05), with the highest concentrations detected in cattle fed high-NFC/NDF diets supplemented with allicin. Serum uric acid was elevated by high NFC/NDF (*p* < 0.05). High dietary NFC/NDF also increased urine pH (*p* < 0.05). Significant interactions between dietary NFC/NDF and allicin supplementation were observed for allantoin, creatinine, and urease activity (*p* < 0.05). High NFC/NDF increased N excretion, total N excretion, and N excretion rate (*p* < 0.01). Additionally, allicin supplementation reduced fecal N excretion and N excretion rate and increased the apparent total-tract digestibility of N (*p* < 0.05). Fecal microbiota Alpha diversity analysis showed that high NFC/NDF decreased the Chao1, observed features, and Shannon (*p* < 0.05). At the phylum level, high NFC/NDF increased Pseudomonadota but decreased Cyanobacteriota, and a significant interaction between NFC/NDF and allicin was observed for Spirochaetota (*p* < 0.05). At the genus level, high NFC/NDF reduced *unclassified_UCG-010* but increased *Succinivibrio*, *unclassified_Muribaculaceae*, and *Methanobrevibacter* (*p* < 0.05). Allicin increased *Rikenellaceae_RC9_gut_group* abundance (*p* < 0.05). In conclusion, dietary NFC/NDF and allicin supplementation modulate acid–base balance, N utilization, and fecal microbial composition in beef bulls, providing a scientific basis for improving feed efficiency.

## Simple Summary

Efficient feeding of beef cattle is essential for both animal growth and environmental sustainability. This study examined how the dietary balance between non-fibrous carbohydrates (NFC) and neutral detergent fiber (NDF), together with the addition of allicin, a natural compound from garlic, affects cattle health, nitrogen emissions, and gut bacterial communities. Forty bulls were fed diets with different NFC/NDF, with or without allicin supplementation, for 75 days. We found that diets with higher NFC/NDF altered blood acidity, increased feces N excretion and N excretion rate and changed the composition of gut bacteria, which could lead to higher environmental N excretion. Allicin supplementation helped reduce N excretion and influenced certain blood and urine markers. Both the dietary NFC/NDF and allicin supplementation affected the diversity and composition of gut microbiota, which play important roles in digestion and overall health. These findings suggest that optimizing the NFC/NDF in diets and supplementing with allicin can improve N utilization, promote cattle health, and mitigate environmental impacts, providing a feeding strategy for sustainable beef production.

## 1. Introduction

Dietary carbohydrates provide approximately 75–85% of the energy required by animals and play a critical role in maintaining growth, metabolism, and production performance in cattle. The structural composition of dietary carbohydrates is therefore an important factor influencing both growth and health in cattle [1]. Carbohydrates in the diet can be broadly categorized into fibrous carbohydrates (FC) and non-fibrous carbohydrates (NFC). Fibrous carbohydrates primarily function to maintain rumen health while also serving as an energy source for the animal. In contrast, NFC—mainly composed of starch, sucrose, and pectin—provides readily fermentable energy for rumen microorganisms and contributes to the host’s energy supply, with glucose production as a secondary outcome [2]. Neutral detergent fiber (NDF) is an important indicator used to evaluate the fiber level in ruminant diets. NDF contributes to rumen fill, determines satiety, and is negatively correlated with feed intake [3]. Its concentration strongly influences the normal function and health of the rumen [4]. Increasing dietary starch or NFC concentrations has been reported to enhance milk production in dairy cows and improve the utilization of energy and nitrogen (N) in ruminants [5]. Nevertheless, excessively high NFC/NDF may impair rumen health by altering fermentation patterns, increasing the risk of subacute ruminal acidosis and ruminal epithelial inflammation [2]. Previous studies have also shown that optimizing the NFC/NDF can improve gross energy digestibility while simultaneously reducing methane production, methane yield, methane intensity, and the methane conversion factor [6]. Methane emissions from livestock represent a major contributor to the accumulation of greenhouse gases in the atmosphere, accounting for approximately 80% of agricultural greenhouse gas emissions and contributing about 15–20% of the overall impact on global warming [7]. The standards issued by the National Research Council (NRC, 2001) provide recommended levels for balancing carbohydrate structure in diets for lactating dairy cows [8]. However, the optimal NFC/NDF for beef cattle has not yet been clearly established. Determining an appropriate ratio is therefore of considerable importance for improving feed utilization efficiency and mitigating greenhouse gas emissions in beef production systems.

Allicin, chemically known as diallyl thiosulfinate (C_6_H_10_S_2_O), is composed of garlic-derived compounds including allicin derivatives and various allyl sulfide compounds. It is a pale yellow oily liquid with a strong pungent odor, insoluble in water but readily soluble in organic solvents such as ethanol, chloroform, and ether [9]. Growth-promoting effects of allicin have been demonstrated in both pigs and sheep [10,11]. Previous studies also suggest that allicin may inhibit the synthesis of membrane lipids in archaeal communities, thereby reducing the population of methanogenic archaea and ultimately decreasing methane emissions [12]. Additionally, garlic-derived products have shown considerable potential to improve rumen energy distribution and enhance ruminal microbial protein synthesis [13]. Recent studies have further demonstrated that allicin supplementation in high-concentrate diets contributes to the maintenance of rumen health [14].

To date, the combined effects of dietary NFC/NDF and allicin supplementation have not been systematically investigated. We hypothesized that an increased NFC/NDF ratio would decrease N utilization efficiency in beef bulls, whereas allicin supplementation would have a beneficial effect on N utilization. Therefore, the present study investigated the effects of allicin supplementation under different NFC/NDF levels on serum electrolytes, nitrogen and phosphorus excretion, and fecal microbiota in beef cattle. The results are expected to provide practical guidance for improving growth performance and reducing emissions in the livestock industry.

## 2. Materials and Methods

### 2.1. Experimental Design

The experiment was conducted from April to July 2025 at Wanchi Breeding Farm, Zhaohua District, Guangyuan City, China. A total of 40 healthy Simmental crossbred beef bulls in the growing–finishing stage (12 ± 0.5 months of age; 293 ± 23.7 kg body weight) were selected and randomly assigned to four treatment groups in a 2 × 2 factorial design. This design evaluated two non-fibrous carbohydrate to neutral detergent fiber ratios (NFC/NDF) (1.02 and 0.75) and two allicin supplementation levels (0 and 0.04% on a DM basis): NFC/NDF 1.02, NFC/NDF 0.75, NFC/NDF 1.02 + 0.04% allicin, and NFC/NDF 0.75 + 0.04% allicin. The NFC/NDF levels were selected based on the forage-to-concentrate ratios commonly used in practical production systems. The supplementation dosage of allicin was determined according to previous studies [15,16] and the manufacturer’s recommendations. Each group included 10 animals, with one animal per replicate. Allicin used in this study was produced by Henan Kangxu Biotechnology Co., Ltd., (Zhengzhou, China), with a purity of 97% (C_6_H_10_S_2_O). Experimental diets were formulated according to the Nutrient Requirements of Beef Cattle to meet the nutritional requirements of finishing cattle weighing 300 kg and targeting an average daily gain of 1.5 kg/d [17]. The ingredient composition and nutrient levels of the diets are presented in Table 1.

Prior to the experiment, all animals were vaccinated, dewormed, and housed in disinfected pens. Allicin was first thoroughly mixed with the concentrate as a feed additive and then incorporated into the total mixed ration (TMR) before feeding. During the experimental period, cattle were fed the TMR twice daily at 06:30 and 16:30, with ad libitum access to feed and water. Feed refusals were maintained at approximately 5%. The entire experimental period lasted 75 days, including a 15-day adaptation period followed by a 60-day formal experimental period.

### 2.2. Sample Collection

On day 60 of the experiment, blood samples were collected from the tail vein of each animal before the morning feeding following an overnight fast. Samples were collected into anticoagulant-free vacuum tubes, kept at 4 °C for 1 h, and centrifuged at 3000 r/min for 10 min. The separated serum was transferred into sterile 2 mL centrifuge tubes and stored at −80 °C until analysis of serum biochemical parameters and electrolyte/acid–base indices.

Fresh fecal samples were collected from each beef cattle over the final five consecutive days of the experimental period, and pH was measured immediately each day. All fecal samples from each cow were then thoroughly homogenized and divided into two portions. One portion (400 g) was acidified with 20% (*v*/*v*) hydrochloric acid at a ratio of 5 mL per 100 g of feces, thoroughly mixed, sealed, and stored at −20 °C for the determination of fecal nitrogen (N). The remaining portion (600 g) was sealed in plastic bags and stored at −20 °C for the analysis of other nutritional components. On the final day of the experiment, fresh fecal samples were collected directly from the rectum of each animal using sterile disposable gloves. To avoid cross-contamination, a new pair of gloves was used for each animal, and samples were transferred into sterile collection tubes immediately after sampling. The samples were then snap-frozen in liquid nitrogen and stored at −80 °C for subsequent microbiota analysis.

Urine samples were collected over the same five-day period, and pH was measured immediately each day. The urine samples from each animal were then pooled, thoroughly mixed, and divided into two subsamples. A 50 mL aliquot was transferred into a sample tube, sealed, labeled, and stored at −20 °C for the determination of urinary physiological and biochemical parameters. An additional 500 mL of urine was placed in a sample bottle and acidified with 20% (*v*/*v*) hydrochloric acid at 14% of the urine volume to reduce the pH to <3. These samples were then sealed and stored at −20 °C for the determination of urinary N.

### 2.3. Chemical Analysis

Serum levels of creatinine (CREA) and uric acid (UA) were analyzed using an automated biochemical analyzer (BS460, Mindray Bio-Medical Electronics Co., Ltd., Shenzhen, China). CREA and UA were measured using the sarcosine oxidase [18] and uricase–peroxidase [19] methods, respectively.

Serum electrolyte and acid–base parameters were measured using a Cobas-b-123 system (Roche Diagnostics International Ltd., Basel, Switzerland) (an ABG analyzer), including potassium (K), sodium (Na), chloride (Cl), ionized calcium (iCa), non-ionized calcium (nCa), total calcium (TCa), pH, carbon dioxide (CO_2_), and anion gap (AG).

Urinary CREA, UA, and urease activity (UE) were determined using an automated biochemical analyzer (BS360S, Mindray Bio-Medical Electronics Co., Ltd., Shenzhen, China) with Mindray commercial reagent kits, following the manufacturer’s protocols. Urinary allantoin (All) concentration was measured using an ELISA kit (Jiangsu Enzyme Immunoassay Co., Ltd., Yancheng, China). Urinary ammonia nitrogen (NH_3_-N) concentration was determined using the distillation–neutralization titration method according to National Environmental Protection Standard of the People’s Republic of ChinaHJ 537-2009 (2010).

The nutrient composition of feed, fecal, and urine samples was determined following the methods of Zhao et al. [20]. Briefly, feed and fecal samples were dried at 65 °C for 48 h, weighed, and ground through a 1 mm sieve. DM and ash were determined according to AOAC (2005; Methods 930.15 and 942.05). N was analyzed by the Kjeldahl method (AOAC 981.10) and expressed as crude protein (CP; N × 6.25). Ether extract (EE) was determined by Soxhlet extraction (AOAC 920.39). Calcium (Ca) and phosphorus (P) were determined by atomic absorption spectrometry and fluorescence spectrophotometry, respectively (AOAC 985.35 and 986.24). Neutral detergent fiber (NDF) and acid detergent fiber (ADF) were analyzed according to Van Soest et al. [21]. Acid-insoluble ash (AIA) was determined according to the method of Van Keulen et al. [22].

### 2.4. Calculation of Nitrogen and Phosphorus Excretion

The N and P emissions of feces from the experimental cattle were estimated using AIA as an endogenous indicator. Based on the AIA concentrations in the diet and feces, daily fecal dry matter (FDM) excretion was estimated, and fecal N and P excretion were subsequently calculated [23]:

FDM (kg/d) = DMI × (AIA_feces_/AIA_diet_)

Daily fecal N (N_feces_) and P excretion (P_feces_) (g/d):

N_feces_ (g/d) = FDM (kg/d) × N_feces_ (g/kg, DM)

P_feces_ (g/d) = FDM (kg/d) × P_feces_ (g/kg, DM)

And daily urine volume (UV) was estimated using the creatinine clearance rate method based on serum/plasma creatinine (Pcr), urinary creatinine (Ucr), and body weight (BW), which was then used to calculate daily urinary N (N_urine_) and P (P_urine_) excretion [24]:

Creatinine clearance rate (Ccr, mL/min) = (k × BW/Pcr),

where BW is body weight (kg), Pcr is serum/plasma creatinine concentration, and k is the species constant (k ≈ 3.7 mg/L for cattle).

Urine flow rate (V, mL/min) = (Ccr × Pcr)/Ucr

Daily UV (L/d) = V (mL/min) × 60 × 24/1000

N_urine_ (g/d) = UV (L/d) × U_N_ (g/L)

P_urine_ (g/d) = UV (L/d) × U_P_ (g/L)

Total N excretion (N_total_, g/d) = N_feces_ + N_urine_

Total P excretion (P_total_, g/d) = P_feces_ + P_urine_

Then the N (N_excretion_) and P (P_excretion_) excretion rate were calculated as follows:

N_excretion_ (%) = N_totle_/N_intake_ × 100

P_excretion_ (%) = P_totle_/P_intake_ × 100

The apparent total-tract digestibility (ATTD) of nitrogen (N_ATTD_) and phosphorus (P_ATTD_) was determined using the AIA marker method as follows [25]:

N_ATTD_ (%) = 100 − (AIA_diet_/AIA _faces_) × (N_faces_/N_diet_) × 100

P_ATTD_ (%) = 100 − (AIA_diet_/AIA_faces_) × (P_faces_/P_diet_) × 100

### 2.5. Fecal DNA Extraction, 16S RNA Sequencing, and Bioinformatics

According to the manufacturer’s instructions, microbial community genomic DNA was extracted from fecal samples using the Magnetic Soil and Stool DNA Kit (Tiangen Biotech, Beijing, China; Catalog No. DP712). DNA quality and integrity were assessed by 1% agarose gel electrophoresis, and DNA concentration and purity were determined using a NanoDrop 2000 UV–Vis spectrophotometer (Thermo Scientific, Wilmington, NC, USA).

The V3–V4 region of the bacterial 16S rRNA gene sequencing was amplified by polymerase chain reaction (PCR) using the primer pair 341F (5′-CCTAYGGGRBGCASCAG-3′) and 806R (5′-GGACTACNNGGGTATCTAAT-3′). PCR products were purified using a magnetic bead–based purification system. Purified amplicons were pooled in equimolar ratios based on individual sample concentrations and thoroughly homogenized. Sequencing libraries were constructed following the standard protocol of Novogene (Beijing, China). Library quality was assessed using Qubit fluorometry and quantitative PCR (qPCR). Following quality validation, the libraries were sequenced on an Illumina NovaSeq 6000 platform (Illumina, San Diego, CA, USA).

Raw sequencing data were demultiplexed based on barcode sequences and PCR primer information, allowing separation of reads from different samples. After removal of barcode and primer sequences, paired-end reads were merged using FLASH software (Version 1.2.11) to generate raw merged sequences (Raw Tags). Residual primer sequences were further removed using Cutadapt to minimize interference in downstream analyses. The Raw Tags were subjected to stringent quality filtering using fastp software (Version 0.23.1), during which low-quality reads, sequences containing ambiguous bases, and short reads were discarded to obtain high-quality sequences (Clean Tags). Chimeric sequences were identified and removed by comparison against the SILVA reference database (https://www.arb-silva.de/), (1 August 2025) resulting in high-confidence representative sequences. The Effective Tags were then processed using the DADA2 plugin in the QIIME2 platform for denoising, including error correction, dereplication, and chimera removal, yielding Amplicon Sequence Variants (ASVs) and an ASV feature table. Taxonomic classification of 16S rRNA gene sequences was performed using the SILVA database (release 138.1).

Alpha diversity indices, including observed ASVs, Shannon, Simpson, and Chao1 indices, were calculated using QIIME2. Beta diversity was assessed based on weighted and unweighted UniFrac distance metrics. Venn diagrams of ASVs, principal coordinates analysis (PCoA), and taxonomic composition bar plots of fecal microbiota were generated using R software (version 3.6.0).

### 2.6. Statistical Analysis

Statistical analyses were performed using SPSS 17.0 software (SPSS Inc., Chicago, IL, USA) for a 2 × 2 factorial design. All data were analyzed by analysis of variance (ANOVA) using the GLM procedure to assess the effects of dietary NFC/NDF, allicin supplementation, and their interaction. Results were presented as means and standard error of the mean. Differences were considered highly significant at *p* < 0.01 and significant at *p* < 0.05.

## 3. Results

### 3.1. Serum Electrolytes and Acid–Base Balance

The effects of dietary NFC/NDF and allicin supplementation on serum electrolytes and acid–base balance in beef bulls were presented in Table 2. The dietary NFC/NDF significantly affected serum pH, with the serum pH in the high NFC/NDF diet being significantly lower than that in the low NFC/NDF diet (*p* < 0.05). In contrast, the AG was higher in the high NFC/NDF diet than in the low NFC/NDF diet (*p* < 0.05). The concentrations of nCa and TCa were significantly influenced by the interaction between dietary NFC/NDF and allicin supplementation (all *p* < 0.05). The highest values were observed in the high NFC/NDF diet supplemented with allicin, whereas the lowest values were found in the low NFC/NDF diet supplemented with allicin. However, serum concentrations of K, Na, Cl, iCa, and CO_2_ were not significantly affected by either dietary NFC/NDF or allicin supplementation (*p* > 0.05).

### 3.2. Serum Creatinine and Uric Acid

The effects of dietary NFC/NDF and allicin supplementation on serum creatinine and uric acid concentrations in beef bulls were presented in Table 3. The serum UA concentration was significantly higher in cattle fed the high NFC/NDF diet than in those fed the low NFC/NDF diet (*p* < 0.01).

### 3.3. Urinary Parameters

The effects of dietary NFC/NDF and allicin supplementation on urinary metabolites in beef bulls are summarized in Table 4. Urine pH was significantly increased by a high dietary NFC/NDF (*p* < 0.05). Significant interactions between dietary NFC/NDF and allicin supplementation were observed for All, CREA, and UE (*p* < 0.05). The highest All concentration was observed in cattle fed a high NFC/NDF diet, whereas the lowest concentration occurred in the low NFC/NDF diet with allicin supplementation. CREA and UE concentrations were elevated in cattle receiving a high NFC/NDF diet supplemented with allicin. In contrast, UA concentration and NH_3_-N were not significantly influenced by either dietary NFC/NDF or allicin supplementation (*p* > 0.05).

### 3.4. Nitrogen and Phosphorus Excretion

The effects of dietary NFC/NDF and allicin supplementation on nitrogen and phosphorus excretion in beef bulls are presented in Table 5. A high dietary NFC/NDF significantly increased fecal N excretion, total N excretion, and N excretion rate (*p* < 0.01), and it decreased ATTD of N (*p* < 0.01). In contrast, allicin supplementation significantly reduced fecal N excretion, total N excretion, and N excretion rate (*p* < 0.05), thereby improving the ATTD of N (*p* < 0.01). However, P excretion was not significantly affected by either dietary NFC/NDF or allicin supplementation (*p* > 0.05).

### 3.5. Fecal Microbial

**Figure 1:**
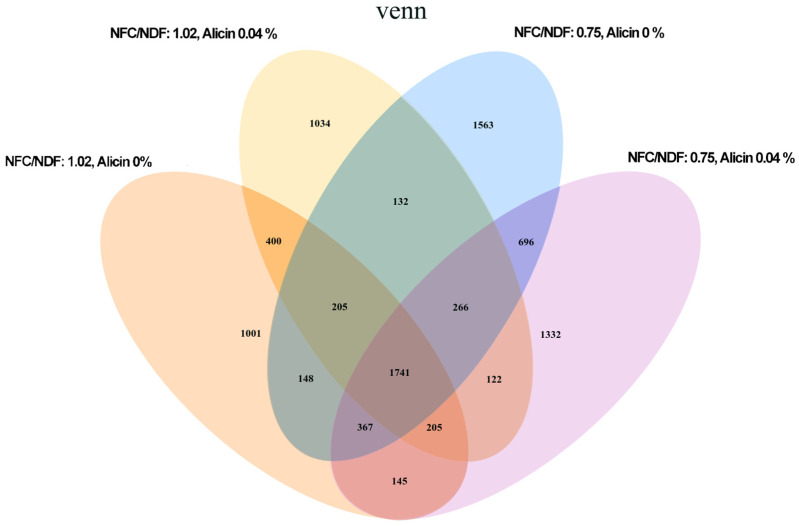
Venn diagram of the Amplicon Sequence Variants (ASVs) in fecal microbiota.

**Figure 2:**
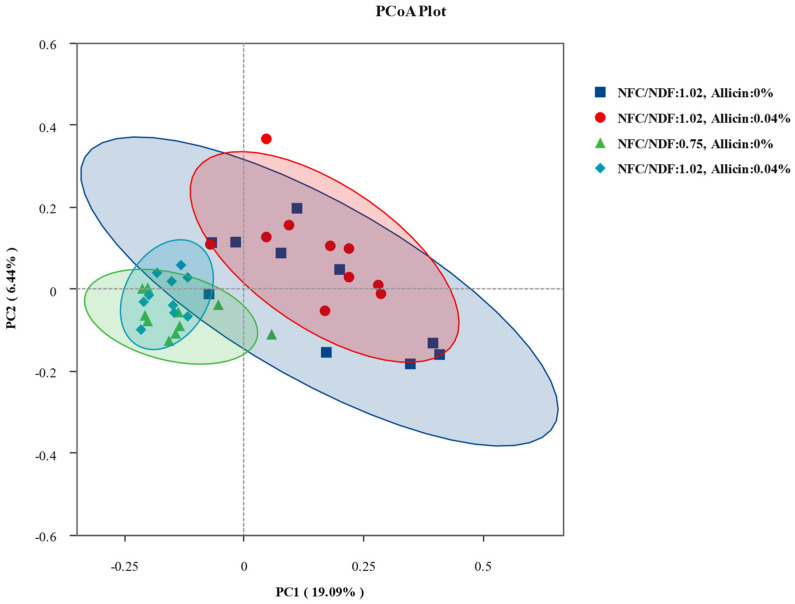
Principal coordinates analysis (PCoA) of fecal microbiota.

The distribution of Amplicon Sequence Variants (ASVs) in fecal microbiota is illustrated in Figure 1. A total of 1741 ASVs were shared among the four groups. The number of unique ASVs, ranked from highest to lowest, was observed in the following order: the low NFC/NDF diet without allicin supplementation group (1563), the low NFC/NDF diet with allicin supplementation group (1332), the high NFC/NDF diet with allicin supplementation group (1034), and the high NFC/NDF diet without allicin supplementation group (1001). As shown in the principal coordinates analysis (PCoA) of fecal microbiota in Figure 2, samples from each group clustered closely together, indicating relatively low inter-individual variability.

The effects of dietary NFC/NDF and allicin supplementation on the fecal microbial α-diversity of beef cattle are presented in Table 6. As shown in Table 6, the Chao1, observed ASVs, and Shannon indices were significantly reduced in the high NFC/NDF diet group compared with the low NFC/NDF group (*p* < 0.05). Furthermore, significant interactions between dietary NFC/NDF and allicin supplementation were observed for the Simpson, Dominance, and Pielou_e indices (*p* < 0.05). The Simpson and Pielou_e indices were highest in the low NFC/NDF group without allicin supplementation and lowest in the corresponding group receiving allicin supplementation. Conversely, the Dominance index showed the opposite trend.

**Figure 3:**
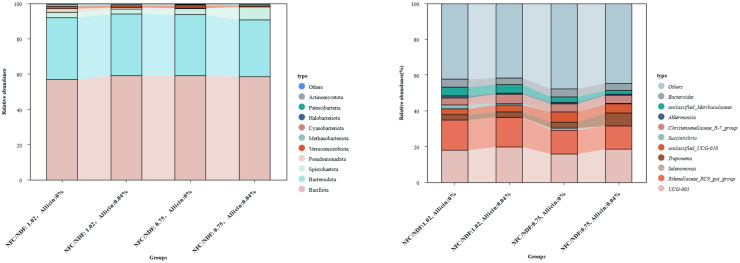
Taxonomic composition of fecal microbiota at the phylum and genus levels (top 10).

As shown in Table 7 and Figure 3, analysis at the phylum level indicated that the dominant bacterial phyla were consistently Bacillota (56.67–59.86%) and Bacteroidota (32.11–34.80%). Neither the main effects of dietary NFC/NDF ratio and allicin supplementation nor their interaction significantly affected the relative abundance of these dominant phyla (*p* > 0.05). A high NFC/NDF significantly increased the relative abundance of Pseudomonadota (*p* < 0.05) while significantly decreasing the relative abundance of Cyanobacteriota (*p* < 0.05). Moreover, a significant interaction between dietary NFC/NDF and allicin supplementation was observed for Spirochaetota (*p* < 0.05). Under allicin supplementation, the relative abundance of Spirochaetota was lowest in the high NFC/NDF group and highest in the low NFC/NDF group.

At the genus level, a high NFC/NDF significantly reduced the relative abundance of *unclassified_UCG-010* (*p* < 0.05), but significantly increased the relative abundance of *Succinivibrio*, *unclassified_Muribaculaceae*, and *Methanobrevibacter* (*p* < 0.05). Allicin supplementation significantly increased the relative abundance of *Rikenellaceae_RC9_gut_group* (*p* < 0.05).

## 4. Discussion

Limited research has investigated the effects of dietary NFC/NDF on serum electrolytes and acid–base balance. Electrolytes play essential roles in maintaining osmotic pressure, regulating acid–base homeostasis, and controlling water metabolism [30]. In the present study, increasing the dietary NFC/NDF ratio resulted in elevated serum AG concentrations and reduced serum pH, whereas other variables affecting acid–base balance remained unaffected. Blood pH, pCO_2_ (a respiratory parameter), bicarbonate concentration, total carbon dioxide, and AG are commonly used indicators for evaluating acid–base status. Deviations of any of these parameters from their physiological ranges may indicate the presence of an acid–base disturbance [31]. Generally, a blood pH ≤ 7.35 is considered indicative of systemic acidosis [32]. Although serum pH decreased with increasing dietary NFC/NDF in the present study, the observed values (7.70–7.75) remained substantially higher than the threshold associated with systemic or metabolic acidosis. These findings suggest that metabolic acidosis was not induced under the experimental conditions. Nevertheless, the simultaneous increase in AG and decrease in pH may indicate a shift toward a more acidogenic metabolic profile. This effect may be attributed to the higher concentration proportion in the high-NFC/NDF diet. Specifically, the concentrate-to-forage ratio was 57:43 in the high-NFC/NDF diet compared with 52:48 in the low-NFC/NDF diet. High-concentrate diets are known to promote the rapid ruminal fermentation of starch and other readily fermentable carbohydrates, resulting in increased production of volatile fatty acids and lactate [33]. Under prolonged feeding conditions, the accumulation of these acidic metabolites may reduce ruminal pH and increase the risk of subacute ruminal acidosis [32]. In severe cases, excessive absorption of lactate and other organic acids from the rumen into the bloodstream may exceed the buffering capacity of systemic regulatory mechanisms, leading to systemic acidification and the development of metabolic acidosis [33,34]. Therefore, although the dietary NFC/NDF ratios evaluated in the present study did not induce metabolic acidosis, maintaining an appropriate NFC/NDF ratio during long-term feeding remains important for preserving ruminal health and sustaining systemic acid–base homeostasis. Furthermore, an interaction between dietary allicin supplementation and dietary NFC/NDF was observed for serum nCa and TCa concentrations. Calcium is not only a major structural component of bones and teeth but also plays critical roles in maintaining normal neuromuscular excitability, regulating muscle contraction, and facilitating blood coagulation. Moreover, calcium is essential for normal cellular physiological functions [35]. Previous studies have shown that dietary allicin supplementation can significantly improve the apparent digestibility of calcium in weaned piglets [10]. However, the mechanisms underlying the interaction between allicin supplementation and dietary NFC/NDF on calcium metabolism remain unclear and warrant further investigation.

Dietary NFC/NDF and allicin supplementation also influenced N metabolism in beef cattle. An increase in the dietary NFC/NDF resulted in higher fecal N, total N excretion, and N excretion rate, indicating a reduction in N utilization efficiency. Meanwhile, the reduction in urinary NH_3_ observed with increasing dietary NFC/NDF may be associated with increased fecal N excretion, indicating that a greater proportion of N was lost in the gastrointestinal tract prior to absorption into the systemic circulation. Consequently, less N would have been available for post-absorptive metabolism and urinary excretion [36]. The decrease in urine pH may be attributed to alterations in systemic acid–base balance resulting from changes in ruminal fermentation associated with higher NFC/NDF conditions. Previous studies investigating the effects of dietary NFC/NDF on N utilization have yielded inconsistent results. For example, Zhou et al. [37] reported that ewes fed a diet with an NFC/NDF ratio of 2.17 exhibited greater ATTD of crude protein than those fed a diet with an NFC/NDF ratio of 0.78. Similarly, Dong et al. [38] found that replacement heifers receiving a diet with an NFC/NDF ratio of 1.64 had higher ATTD of crude protein than those fed diets with NFC/NDF ratios of 1.36 or 1.12. In contrast, no significant differences in crude protein digestibility were observed between diets with NFC/NDF ratios of 1.71 and 1.07 in castrated Holstein steers [2]. Likewise, Ma et al. reported no difference in the ATTD of crude protein between diets with NFC/NDF ratios of 1.0 and 1.7 [39]. However, ruminal crude protein digestibility was significantly reduced, whereas post-ruminal crude protein digestibility was significantly increased under the high NFC/NDF diet. In the present study, the high NFC/NDF group (1.02) exhibited significantly lower ATTD of N compared with the low NFC/NDF group (0.75). These discrepancies may be largely attributed to differences in diet formulation across studies. In previous work reporting increased crude protein digestibility with higher NFC/NDF, dietary NFC/NDF was primarily manipulated by altering the concentrate-to-forage ratio, without strict control of dietary crude protein or metabolizable energy levels. Therefore, the improved N digestibility observed in those studies may have been confounded by higher dietary energy or protein supply [37,38]. In contrast, dietary energy and crude protein contents were carefully balanced between treatments in the present study. Moreover, a relatively large proportion of corn was included in the present diets to increase dietary NFC, which may have accelerated ruminal fermentation. Previous studies have suggested that high-NFC diets are associated with an increased digesta passage rate compared with high-NDF diets, potentially reducing the efficiency of ruminal N digestibility [39]. This mechanism may partially explain the reduced N digestibility observed in the present study. N utilization depends not only on the amount of fermentable carbohydrates but also on the synchrony between energy and nitrogen availability in the rumen [40]. The low NFC/NDF diet may have promoted a more synchronized release of fermentable substrates and N, thereby improving microbial N utilization efficiency [41]. Furthermore, the reduction in total N excretion observed in the present study was primarily driven by decreased fecal N excretion, whereas urinary N excretion was not significantly affected. These findings suggest that differences in N utilization were mainly associated with digestive processes rather than post-absorptive metabolism under the physiological conditions of beef bulls. Therefore, it is speculated that the higher fiber content in the low NFC/NDF diet may have stimulated chewing activity, enhanced intestinal motility, and altered digesta passage characteristics, which may have contributed to improved nutrient digestibility [42]. Finally, soybean oil was included in the low NFC/NDF diet to maintain comparable dietary energy density between treatments in the present study; therefore, the potential contribution of supplemental lipids to nitrogen utilization cannot be fully excluded [43]. In addition, serum CREA concentrations were decreased in the high NFC/NDF group in the present study, whereas urinary CREA concentrations remained unchanged. As CREA is primarily produced through muscle creatine metabolism and eliminated via glomerular filtration, these findings suggest that the observed changes are more likely attributable to altered CREA production or muscle metabolic activity, rather than differences in renal clearance [44]. It has been reported that blood CREA concentrations are positively associated with average daily gain in animals [45]. Consistently, Lawrence et al. [46] observed higher CREA levels in more efficient cattle characterized by lower residual feed intake, indicating that improved feed efficiency may be accompanied by elevated CREA concentrations. Collectively, these findings support the inference that a higher NFC/NDF may reduce N utilization efficiency in beef cattle.

Furthermore, the present results showed that dietary supplementation with allicin reduced fecal N excretion, total N excretion, and N excretion rate, thereby improving ATTD of N. Several recent studies have reported similar findings. For example, Guo et al. demonstrated that supplementation with 400 g of allicin improved ATTD of crude protein digestibility in Small-tailed Han sheep [11]. Likewise, Chao et al. reported that supplementation with 0.75 g/d of allicin significantly increased ATTD of crude protein in goats [13]. These effects may be associated with alterations in gastrointestinal microbial populations induced by allicin supplementation.

The effects of dietary NFC/NDF and allicin supplementation on fecal microbiota in beef cattle were investigated. The results of the present study further demonstrated that high dietary NFC/NDF reduced microbial richness in cattle feces. Similar findings have been reported in ruminants, where reduced rumen bacterial diversity has been associated with improved feed efficiency [30]. In previous studies, high-NFC diets increased ruminal microbial diversity and bacterial richness in sheep compared with low-NFC diets [47], whereas high-concentrate feeding has been shown to reduce rumen microbial richness and diversity, leading to decreased relative abundance of Bacteroidetes. Recent studies have increasingly focused on dietary allicin supplementation. Yuan et al. reported that appropriate levels of allicin increased microbial richness in the rumen, while richness in the jejunum and cecum decreased [15]. Furthermore, allicin supplementation in high-concentrate diets has been shown to enhance rumen microbial diversity [16]. Similarly, in the present study, an interactive effect of dietary NFC/NDF and allicin on fecal microbial diversity was observed. At the phylum level, Firmicutes and Bacteroidetes are the dominant taxa in healthy ruminant rumens, with Firmicutes primarily responsible for fiber degradation and volatile fatty acid production, which serve as energy sources for the host [48,49]. In the present study, dietary NFC/NDF and allicin supplementation did not alter the relative abundance of these dominant phyla. High NFC/NDF diets increased the relative abundance of Pseudomonadota but decreased that of Cyanobacteriota. Previous studies have demonstrated that an increased abundance of Pseudomonadota is closely associated with a higher incidence of diarrhea in cattle [50,51]. The increased relative abundance of Pseudomonadota observed in the high NFC/NDF group in the present study is consistent with the findings of Li et al. [52], who reported that a high dietary NFC/NDF ratio promotes the proliferation of potentially harmful bacteria, including Pseudomonadota. In addition, diets with a high NFC/NDF contain higher levels of rapidly fermentable carbohydrates, mainly from corn-derived starch, which can disrupt microbial balance, induce intestinal inflammation, and subsequently lead to diarrhea [52]. Cyanobacteriota are known to participate in various physiological processes in the intestine, including vitamin B synthesis, anaerobic fermentation, oxygen-dependent hydrogen production, nitrogen fixation, the degradation of hemicellulose and pectin, and methane mitigation [53]. The reduced abundance of Cyanobacteriota observed in the high NFC/NDF group may therefore be associated with increased N excretion. In addition, the present study identified an interaction effect between dietary NFC/NDF and allicin supplementation on the abundance of Spirochaetota. Spirochaetota has been reported to harbor multiple carbohydrate-degrading genes in swine [54] and is involved in the hydrolysis of complex plant polysaccharides, protein degradation, and vitamin B synthesis in the rumen, potentially influencing protein and fiber digestibility [55]. At the genus level, a high dietary NFC/NDF reduced the relative abundance of *unclassified_UCG-010*. This finding is consistent with previous studies, in which high-concentrate diets were shown to decrease *unclassified_UCG-010* in the colon of Hu sheep and induce barrier damage [56]. *Unclassified_UCG-010*, belonging to the family *Ruminococcaceae*, is primarily involved in starch and fiber degradation in ruminants and contributes to further fermentation of feed within the gastrointestinal tract. In contrast, the relative abundances of *Succinivibrio*, *unclassified_Muribaculaceae*, and *Methanobrevibacter* were significantly increased in the high NFC/NDF group. *Succinivibrio* has been positively correlated with average daily gain in sheep and is associated with enhanced carbohydrate fermentation capacity and improved host energy supply [57]. *Unclassified_Muribaculaceae* has been shown to regulate butyrate concentrations, thereby influencing intestinal barrier function and inflammation [58]. *Methanobrevibacter*, a dominant methanogenic archaeon responsible for converting fermentation-derived hydrogen into methane [59], was enriched in animals fed the higher NFC/NDF diet, potentially due to the increased availability of rapidly fermentable substrates. Previous studies have generally reported that increasing the dietary NFC/NDF ratio reduces methane yield [6,38], a response that is often associated with improved feed efficiency and N utilization [60]. Therefore, the present findings appear to differ from those previously reported. However, most studies have focused on ruminal methanogenesis, whereas the present study characterized fecal microbial communities. Furthermore, methane emissions were not directly measured in this study, and methanogen abundance does not necessarily correlate with actual methane production. The underlying mechanisms linking fecal *Methanobrevibacter* abundance to N utilization require further investigation. *Rikenellaceae_RC9_gut_group*, a member of the *Rikenellaceae* family, degrades both soluble polysaccharides and insoluble cellulose; its abundance in feces was increased by allicin supplementation [61].

## 5. Conclusions

This study demonstrated that dietary NFC/NDF and allicin supplementation significantly modulate acid–base balance, N utilization efficiency, and fecal microbial composition in beef bulls. Under the conditions of the present experiment, increasing the dietary NFC/NDF was associated with decreased serum pH, increased serum anion gap and urine pH, and elevated urinary NH_3_ and fecal N excretion, resulting in a higher overall N excretion rate. Allicin supplementation exhibited interactive effects with dietary NFC/NDF on serum calcium concentrations and urinary biochemical parameters, effectively reducing N excretion rate. These alterations were closely linked to shifts in fecal microbial community structure; however, it is noteworthy that both high NFC/NDF and allicin supplementation reduced microbial diversity. These findings provide a scientific basis for improving N utilization efficiency.

## Figures and Tables

**Table 1:** Ingredients and nutrient levels of diets (%, DM basis).

Ingredient	NFC/NDF = 1.02	NFC/NDF = 0.75
Corn	33.37	20.38
Soybean meal	8.01	8.63
Rapeseed meal	4.40	4.40
Wheat bran	6.96	12.96
Corn straw silage	25.74	25.74
Rice straw	17.34	22.32
CaCO_3_	1.26	1.30
CaHPO_4_	0.27	0.12
Salt	0.90	0.90
Premix ^1^	0.95	0.95
NaHCO3	0.80	0.80
Soybean oil	0.00	1.50
Total	100.00	100.00
Nutritional composition ^2^, %		
CP	12.40	12.60
NE, Mcal/kg	3.07	3.02
EE	2.08	3.05
NDF	37.34	42.13
ADF	23.91	27.21
Ash	10.27	10.75
Ca	0.89	0.90
P	0.44	0.43
NFC ^3^	38.12	31.46
NFC/NDF	1.02	0.75

NFC/NDF = non-fibrous carbohydrate to neutral detergent fiber ratio; NE = net energy; CP = crude protein; EE = ether extract; NDF = neutral detergent fiber; ADF = acid-wash fiber; Ca = calcium; P = phosphorus; NFC = non-fibrous carbohydrate. ^1^ The premix was formulated to provide, per kilogram of diet, vitamin A (8800 KIU), vitamin D_3_ (11,000 KIU), vitamin E (240 KIU), magnesium oxide (4 g), ferrous sulfate (2000 mg), zinc sulfate (2500 mg), copper sulfate (500 mg), manganese sulfate (1500 mg), calcium iodate (40 mg), sodium selenite (30 mg), and cobalt chloride (20 mg). ^2^ NFC and NE were calculated values; all other nutrient components were analyzed. ^3^ NFC was calculated using the formula: NFC = 100 − (CP + NDF + EE + Ash) [6].

**Table 2:** Effects of dietary NFC/NDF and allicin supplementation on serum electrolytes and acid–base balance in beef bulls (mmol/L, except for pH).

Item	Allicin (0%)		Allicin (0.04%)		SEM	*p*-Value			Reference Value
	NFC/NDF = 1.02	NFC/NDF = 0.75	NFC/NDF = 1.02	NFC/NDF = 0.75		NFC/NDF	Allicin	NFC/NDF × Allicin	
K	5.16	4.78	4.91	4.90	0.060	0.109	0.568	0.122	3.9–5.8 [26]
Na	140.89	139.59	140.48	140.41	0.290	0.247	0.730	0.300	132–152 [26]
Cl	100.62	100.57	100.00	101.32	0.262	0.232	0.902	0.198	95–110 [26]
iCa	1.07	1.09	1.14	2.33	0.325	0.358	0.322	0.374	--
nCa	1.24	1.27	1.30	1.22	0.012	0.311	0.983	0.018	--
Tca	2.41	2.48	2.52	2.37	0.024	0.343	0.965	0.017	2–2.85 [26]
pH	7.72	7.74	7.70	7.75	0.007	0.011	0.638	0.367	6.9–7.8 [27]
CO_2_	16.34	16.56	16.33	15.66	0.217	0.611	0.306	0.317	--
AG	23.92	22.43	24.13	23.47	0.239	0.021	0.170	0.359	19–26 [28]

NFC/NDF = non-fibrous carbohydrate to neutral detergent fiber ratio; K = potassium; Na = sodium; Cl = chloride; iCa = ionized calcium; nCa = non-ionized calcium; TCa = total calcium; pH = potential of hydrogen; CO_2_ = carbon dioxide; AG = anion gap.

**Table 3:** Effects of dietary NFC/NDF and allicin supplementation on serum creatinine and uric acid concentrations in beef bulls.

Item	Allicin (0%)		Allicin (0.04%)		SEM	*p*-Value			Reference Value
	NFC/NDF = 1.02	NFC/NDF = 0.75	NFC/NDF = 1.02	NFC/NDF = 0.75		NFC/NDF	Allicin	NFC/NDF × Allicin	
CREA, μmol/L	89.92	95.26	94.97	101.18	1.658	0.078	0.094	0.892	88–177 [26]
UA, μmol/L	72.41	53.53	71.46	61.88	1.968	0.000	0.254	0.154	49.67–171.33 [29]

NFC/NDF = non-fibrous carbohydrate to neutral detergent fiber ratio; CREA = creatinine; UA = uric acid.

**Table 4:** Effects of dietary NFC/NDF and allicin supplementation on urinary components in beef bulls.

Item	Allicin (0%)		Allicin (0.04%)		SEM	*p*-Value		
	NFC/NDF = 1.02	NFC/NDF = 0.75	NFC/NDF = 1.02	NFC/NDF = 0.75		NFC/NDF	Allicin	Allicin × NFC/NDF
pH	8.248	8.513	8.357	8.528	0.101	0.038	0.545	0.646
All, μg/mL	129.8	116.2	111.9	112.9	3.028	0.043	0.001	0.021
CREA, μmol/L	2891	3659	4039	2946	395.231	0.684	0.586	0.024
UE, U/mL	0.079	0.144	0.134	0.058	0.034	0.872	0.657	0.046
UA, μmol/L	705.8	577.3	1031.7	589.4	186.289	0.134	0.370	0.405
NH_3_-N, %	0.043	0.097	0.062	0.07	0.018	0.091	0.820	0.199

NFC/NDF = non-fibrous carbohydrate to neutral detergent fiber ratio; All = allantoin; CREA = creatinine; UE = urease activity; UA = uric acid; NH_3_-N = ammonia nitrogen.

**Table 5:** Effects of dietary NFC/NDF and allicin supplementation on nitrogen and phosphorus excretion in beef bulls.

Item	Allicin (0%)		Allicin (0.04%)		SEM	*p*-Value		
	NFC/NDF = 1.02	NFC/NDF = 0.75	NFC/NDF = 1.02	NFC/NDF = 0.75		NFC/NDF	Allicin	Allicin × NFC/NDF
Feces, (DM, kg/d)	3.49	3.35	3.30	3.16	0.102	0.179	0.070	0.997
N_intake_, g/d	183.57	179.13	183.10	175.40	4.914	0.225	0.672	0.742
N_feces_, g/d	89.19	75.30	82.57	69.08	2.689	<0.001	0.022	0.941
N_urine_, g/d	32.72	30.36	24.86	30.01	2.815	0.624	0.154	0.191
N_total_, g/d	121.90	105.66	107.43	99.09	4.352	0.008	0.021	0.370
N_excretion_, %	66.50	59.12	58.73	56.21	1.800	0.009	0.005	0.185
N_ATTD,_ %	51.4	57.93	54.90	60.73	0.794	<0.001	<0.001	0.663
P intake, g/d	39.84	38.88	39.71	39.77	1.078	0.678	0.727	0.638
P_feces_, g/d	25.63	25.62	25.84	23.57	1.566	0.470	0.560	0.475
P_urine_, g/d	1.17	0.78	0.72	0.85	0.314	0.686	0.539	0.417
P_total_, g/d	26.80	26.40	26.56	24.42	4.352	0.371	0.432	0.538
P_excretion_, %	67.00	68.08	66.72	61.07	2.674	0.398	0.181	0.216
P_ATTD,_ %	38.83	33.83	35.18	38.36	3.182	0.777	0.889	0.663

**Table 6:** Effects of dietary NFC/NDF and allicin supplementation on fecal microbial α-diversity in beef bulls.

Item	Allicin (0%)		Allicin (0.04%)		SEM	*p*-Value		
	NFC/NDF = 1.02	NFC/NDF = 0.75	NFC/NDF = 1.02	NFC/NDF = 0.75		NFC/NDF	Allicin	Allicin × NFC/NDF
chao1	1089.073	1497.018	1112.386	1443.648	32.233	0.000	0.817	0.556
dominance	0.008	0.004	0.006	0.009	0.001	0.594	0.139	0.007
observed ASVs	1086.100	1493.500	1108.000	1440.200	32.206	0.000	0.809	0.563
pielou_e	0.845	0.879	0.855	0.858	0.003	0.005	0.382	0.015
shannon	8.482	9.260	8.638	8.994	0.060	0.000	0.648	0.088
simpson	0.992	0.996	0.994	0.991	0.001	0.594	0.139	0.007

NFC/NDF = non-fibrous carbohydrate to neutral detergent fiber ratio.

**Table 7:** Effects of dietary NFC/NDF and allicin supplementation on the relative abundance of fecal microbiota at the phylum and genus levels in beef bulls (%).

Item	Allicin (0%)		Allicin (0.04%)		SEM	*p*-Value		
	NFC/NDF = 1.02	NFC/NDF = 0.75	NFC/NDF = 1.02	NFC/NDF = 0.75		NFC/NDF	Allicin	Allicin × NFC/NDF
Phylum								
Bacillota	56.67	58.98	59.86	58.58	1.593	0.754	0.393	0.275
Bacteroidota	34.52	34.77	34.80	32.11	1.624	0.471	0.481	0.384
Spirochaetota	3.57	3.42	1.91	7.20	0.946	0.012	0.282	0.008
Pseudomonadota	2.38	0.25	1.01	0.20	0.507	0.008	0.181	0.214
Verrucomicrobiota	1.21	1.07	0.80	0.52	0.552	0.716	0.402	0.901
Methanobacteriota	1.24	0.76	1.04	0.90	0.182	0.106	0.879	0.350
Cyanobacteriota	0.03	0.32	0.05	0.13	0.085	0.035	0.317	0.195
Genus								
*UCG-005*	17.46	16.11	19.11	18.99	0.707	0.460	0.028	0.534
*Rikenellaceae_RC9_gut_group*	15.99	13.56	16.95	13.15	1.199	0.071	0.872	0.684
*Treponema*	3.53	3.25	3.05	6.63	0.813	0.154	0.211	0.098
*unclassified_UCG-010*	3.44	5.64	3.44	5.21	0.420	0.002	0.711	0.714
*Succinivibrio*	2.28	0.12	0.95	0.13	0.413	0.014	0.258	0.253
*Christensenellaceae_R-7_group*	4.12	4.62	4.69	4.48	0.343	0.767	0.669	0.469
*unclassified_Muribaculaceae*	5.04	3.07	4.81	2.14	0.376	0.000	0.276	0.509
*Bacteroides*	4.10	4.73	3.79	4.11	0.358	0.349	0.276	0.761
*Alistipes*	2.63	2.74	3.02	2.37	0.332	0.618	0.740	0.484
*Prevotellaceae_UCG-003*	1.31	1.80	1.33	1.47	0.334	0.506	0.740	0.716
*unclassified_[Eubacterium]_coprostanoligenes_group*	2.34	3.51	2.52	3.02	0.334	0.084	0.276	0.481
*unclassified_Bacteroidales_RF16_group*	2.02	2.65	1.62	2.24	0.259	0.103	0.325	0.994
*Methanobrevibacter*	1.05	0.74	0.93	0.74	0.136	0.090	0.658	0.668

NFC/NDF = non-fibrous carbohydrate to neutral detergent fiber ratio.

## Data Availability

The original contributions presented in this study are included in the article. Further inquiries can be directed to the corresponding authors.
